# (1*R*,2*R*,3*R*,4*S*,5*S*)-3-Methyl-8-oxa­bicyclo­[3.2.1]oct-6-ene-2,4-diyl diacetate

**DOI:** 10.1107/S1600536811027292

**Published:** 2011-07-23

**Authors:** Viktor A. Tafeenko, Leonid A. Aslanov, Marina V. Proskurnina, Sergei E. Sosonyuk, Dmitrii A. Khlevin

**Affiliations:** a Chemistry Department, Moscow State University, 119991 Moscow, Russian Federation

## Abstract

The mol­ecule of the title compound, C_12_H_16_O_5_, has crystallographically imposed mirror symmetry with the mirror plane passing through the endocyclic O atom and the mid-point of the double bond. In the crystal, mol­ecules are linked by C—H⋯O hydrogen bonds, forming chains running along the *a* axis.

## Related literature

Compounds containing the 8-oxabicyclo­[3.2.1]octane framework have shown broad utility as chiral building blocks for synthesis of polyketides, see: Coste & Gerber-Lemaire (2005 ▶); Meilert *et al.* (2003 ▶); Schwenter & Vogel (2001 ▶); Gerber-Lemaire & Vogel (2003 ▶); Gerber & Vogel (1999 ▶, 2001 ▶); Re *et al.* (2009 ▶); Pascual *et al.* (2004 ▶); Derwick (1998 ▶). For the inhibitory activity of calystegines and other tropane alkaloids against several glycosidase enzymes, see: Asano *et al.* (2000 ▶); Drager (2004 ▶). Several 8-oxabicyclo­[3.2.1] octane derivatives possess moderate anti-HIV activity, see: Montana *et al.* (2009 ▶). For the syntheses of a full set of hybrid *d*- and l-*C*-glycosides and thymine polyoxin C starting with the unsaturated 8-oxabicyclo­[3.2.1]octane framework, see: Gethin & Simpkins (1997 ▶); Hoffmann *et al.* (2001 ▶). For the synthesis of an 8-oxabicyclo­[3.2.1]octane from tetra­chloro­cyclo­propene and furan, see: Batson *et al.* (2004 ▶). For a synthetic approach to 8-oxabicyclo­[3.2.1]octane derivatives based on the reaction of tetra­chloro­cyclo­propene with furan, see: Law & Tobey (1968 ▶). For structures of related 8-oxabicyclo­[3.2.1]octa­nes, see: Kreiselmeier *et al.* (2006 ▶); Hoffmann *et al.* (2001 ▶). For a report of prior research, see: Tafeenko *et al.* (2009 ▶). 
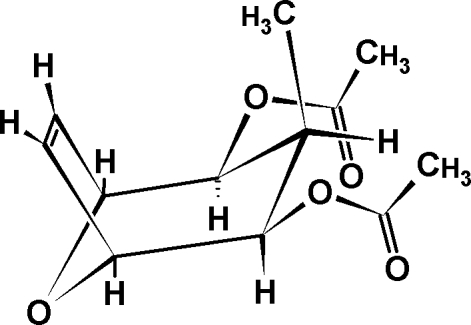


## Experimental

### 

#### Crystal data

C_12_H_16_O_5_
*M* *_r_* = 240.25
Orthorhombic, 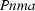

*a* = 6.8680 (12) Å
*b* = 12.295 (4) Å
*c* = 14.120 (3) Å
*V* = 1192.3 (5) Å^3^
*Z* = 4
Ag *K*α radiation
λ = 0.56085 Å
μ = 0.06 mm^−1^
*T* = 296 K
0.1 × 0.07 × 0.05 mm

#### Data collection

Enraf–Nonius CAD-4 diffractometer
1974 measured reflections
1974 independent reflections
1085 reflections with *I* > 2s(*I*)
2 standard reflections every 120 min  intensity decay: none

#### Refinement

*R*[*F* ^2^ > 2σ(*F* ^2^)] = 0.055
*wR*(*F* ^2^) = 0.138
*S* = 1.02
1974 reflections
91 parameters
H atoms treated by a mixture of independent and constrained refinement
Δρ_max_ = 0.24 e Å^−3^
Δρ_min_ = −0.17 e Å^−3^

### 

Data collection: *CAD-4 Software* (Enraf–Nonius, 1989 ▶); cell refinement: *CAD-4 Software*; data reduction: *XCAD4* (Harms & Wocadlo, 1995 ▶); program(s) used to solve structure: *SHELXS97* (Sheldrick, 2008 ▶); program(s) used to refine structure: *SHELXL97* (Sheldrick, 2008 ▶); molecular graphics: *DIAMOND* (Brandenburg, 2000 ▶); software used to prepare material for publication: *WinGX* (Farrugia, 1999 ▶).

## Supplementary Material

Crystal structure: contains datablock(s) I, global. DOI: 10.1107/S1600536811027292/mw2015sup1.cif

Structure factors: contains datablock(s) I. DOI: 10.1107/S1600536811027292/mw2015Isup2.hkl

Additional supplementary materials:  crystallographic information; 3D view; checkCIF report

## Figures and Tables

**Table 1:** Hydrogen-bond geometry (Å, °)

*D*—H⋯*A*	*D*—H	H⋯*A*	*D*⋯*A*	*D*—H⋯*A*
C6—H6⋯O2^i^	0.93	2.55	3.482 (2)	178

Symmetry code: (i) 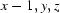.

**Fig. 1.**
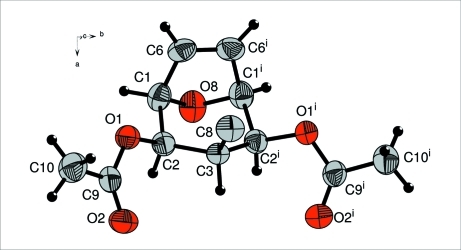
The molecular structure of (I) with displacement ellipsoids drawn at the 50% probability level. Atoms C,N,O, and Ci,Ni,Oi are related by symmetry code: (i) x,1.5 - y, z; The H atoms at C8 are not shown for clarity.

**Fig. 2.**
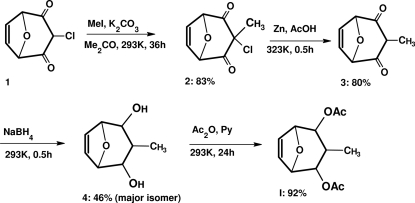
How the title compound was obtained.

## supplementary crystallographic information

### Comment

Compounds containing the 8-oxabicyclo[3.2.1]octane framework are important precursors in the field of biologically active compounds. They have shown broad utility as chiral building blocks for synthesis of polyketides (Coste & Gerber-Lemaire, 2005; Meilert *et al.*, 2003; Schwenter & Vogel, 2001; Gerber-Lemaire & Vogel, 2003), C-linked disaccharides (Gerber & Vogel, 1999; Gerber & Vogel, 2001), calystegines (Re *et al.*, 2009; Pascual *et al.*, 2004; Derwick, 1998) and other natural products. Calystegines and other tropane alkaloids show remarkable inhibitory activities against several glycosidase enzymes, in comparison with other alkaloidal glycosidase inhibitors (Asano *et al.*, 2000; Drager, 2004). They are used medicinally, *e.g.*, as anticholinergics, competing with acetylcholine for the muscarinic receptor site of the parasympathetic nervous system (Derwick, 1998). De novo syntheses of a full set of hybrid d- and l-C-glycosides and thymine polyoxin C starting with the unsaturated 8-oxabicyclo[3.2.1]octane framework have been reported (Hoffmann *et al.*, 2001; Gethin & Simpkins, 1997). Moreover, recent research showed that several 8-oxabicyclo[3.2.1] octane derivatives possess moderate anti-HIV activity (Montana *et al.*, 2009). In studies of novel biologically active homoinositol compounds, including selective glycosidase inhibitors, we have investigated a new synthetic approach to 8-oxabicyclo[3.2.1]octane derivatives based on the reaction of tetrachlorocyclopropene with furan (Law *et al.*, 1968) for the preparation of (1*R*,2*R*,3r,4*S*,5S)-3-methyl-8-oxabicyclo[3.2.1]oct-6-ene- 2,4-diol (4). Several structural results in this field have been previously reported (Tafeenko *et al.*, 2009; Batson *et al.*, 2004; Kreiselmeier *et al.*, 2006; Hoffmann *et al.* 2001).

Determination of the relative stereochemistry of compound (4) (Fig. 2) by NMR methods was ambiguous so recourse was made to X-ray crystallography for which purpose the crystalline diacetate (I) was synthesized.

Molecule (I) has crystallographically-imposed mirror symmetry with the mirror plane, m, passing through atoms C3, C8, the endocyclic oxygen O8 and the midpoint of the double bond C6/C6^i^ (i: *x*,1.5 - *y*, *z*). The 6-membered ring of the molecule adopts a chair conformation, with atoms O8 and C3 displaced out of plane defined by the atoms C2/C2^i^/C1/C1^i^ (plane 1) by 0.856 (2) and -0.525 (2) Å, respectively. The carbon atom of the methyl-group and atoms C6,C6^i^ (double bond) are displaced out of plane 1 by -2.028 (2) Å and -1.326 (2) Å respectively. The molecules (I) are linked by means of weak C—H···O hydrogen bonds to form chains running along *a* axis.

### Experimental

(1*R*,5S)-3-Chloro-3-methyl-8-oxabicyclo[3.2.1]oct-6-ene-2,4-dione (see Fig.2) (2).

To a solution of 0.5 g (2.9 mmol) of (1) (Law & Tobey, 1968) in acetone (10 ml) 0.83 g (5.8 mmol) K_2_CO_3_ and 1.38 g (8.7 mmol) MeI were added. The mixture was stirred at 298 K for 36 h, then concentrated under reduced pressure. The residue was purified *via* silica gel flash chromatography (10% EtOAc in CH_2_Cl_2_), to give a pale yellow crystalline solid; yield: 0.45 g, (83%) (compound 2), mp 370–372 K.

(1*R*,5S)-3-methyl-8-oxabicyclo[3.2.1]oct-6-ene-2,4-dione (see Fig.2) (3).

To a solution of 0.4 g (2.1 mmol) of diketone (2) in 4 ml of glacial acetic acid was added 0.23 g of Zn-powder. After the beginning of heating-up the mixture was stirred at 298 K for 0.5 h and 20% aqueous NaOH was added dropwise until solid formed. The solid was dissolved by addition of 1 ml 0.1 N HCl, the mixture was extracted with CH_2_Cl_2_ (5*20 ml), the combined organic layers were dried over Na_2_SO_4_ and evaporated under reduced pressure to yield 0.26 g of compound (3) as a pale yellow crystalline solid, (compound 3) mp 359–361 K.

(1*R*,2*R*,4*S*,5S)-3-methyl-8-oxabicyclo[3.2.1]oct-6-ene-2,4 -diol (see Fig.2) (4).

To a solution of 200 mg (1.3 mmol) of diketone (3) in 15 ml of MeOH 340 mg (8.9 mmol) of NaBH_4_ was added portionwise for 3 h, during which time the reaction temperature was kept between 293–303 K. The mixture was stirred at 298 K for 2 h, then 0.4 g of crystalline NH_4_Cl was added followed by 0.5 ml of 1 N HCl. The solvents were evaporated under reduced pressure, the residue was flashed by EtOAc/Me_2_CO (1:1) through a silica gel column to give 0.2 g of a yellow oil. The ^1^H and ^13^C NMR spectra showed that the oil contained several isomers so it was purified by column chromatography on silica gel eluting with a EtOAc/Me_2_CO (5:2) mixture to afford 95 mg of the major isomer (4) as a colorless oil, yield 46%; Rf = 0.5 (Et_2_O/Me_2_CO = 3:1).

(1*R*,2*R*,3r,4*S*,5S)-3-methyl-8-oxabicyclo[3.2.1]oct-6-ene- 2,4-diyl diacetate (see Fig.2) (I).

Compound (4) (95 mg, 0.61 mmol) was dissolved in 5 ml of py and 3 ml of Ac_2_O was added. The mixture was stirred for 24 h at room temperature and then concentrated under reduced pressure (1 mm Hg) to give a thick brown oil. Diacetate (I) was separated by flash chromatography on silica gel (CH_2_Cl_2_) as colorless crystals, yield 134 mg (92%), mp 428–430 K, Rf = 0.4 (CH_2_Cl_2_). Crystals suitable for diffraction analysis were obtained by slow evaporation of solvents from a dichloromethane-hexane solution.

### Refinement

The positions of the H atoms were determined from Fourier difference maps; H atoms attached to carbons were then placed in calculated positions and allowed to ride on their parent atoms [C—H = 0.93–0.98 Å. *U*_iso_(H) = *xU*_eq_(parent atom), where *x* = 1.2.] Hydrogen (H8, H81) atoms at C8 are refined freely.

### Crystal data

**Table:**

C_12_H_16_O_5_	*D*_x_ = 1.338 Mg m^−^^3^
*M**_r_* = 240.25	Melting point: 428 K
Orthorhombic, *P**n**m**a*	Ag *K*α radiation, λ = 0.56085 Å
Hall symbol: -P 2ac 2n	Cell parameters from 25 reflections
*a* = 6.8680 (12) Å	θ = 11–14°
*b* = 12.295 (4) Å	µ = 0.06 mm^−^^1^
*c* = 14.120 (3) Å	*T* = 296 K
*V* = 1192.3 (5) Å^3^	Prism, colorless
*Z* = 4	0.1 × 0.07 × 0.05 mm
*F*(000) = 512	

### Data collection

**Table:**

Enraf–Nonius CAD-4 diffractometer	*R*_int_ = 0.000
Radiation source: fine-focus sealed tube	θ_max_ = 24.0°, θ_min_ = 1.7°
graphite	*h* = −9→0
non–profiled ω scans	*k* = −17→0
1974 measured reflections	*l* = −20→0
1974 independent reflections	2 standard reflections every 120 min
1085 reflections with *I* > 2s(*I*)	intensity decay: none

### Refinement

**Table:**

Refinement on *F*^2^	Secondary atom site location: difference Fourier map
Least-squares matrix: full	Hydrogen site location: inferred from neighbouring sites
*R*[*F*^2^ > 2σ(*F*^2^)] = 0.055	H atoms treated by a mixture of independent and constrained refinement
*wR*(*F*^2^) = 0.138	*w* = 1/[σ^2^(*F*_o_^2^) + (0.0492*P*)^2^ + 0.2946*P*] where *P* = (*F*_o_^2^ + 2*F*_c_^2^)/3
*S* = 1.02	(Δ/σ)_max_ < 0.001
1974 reflections	Δρ_max_ = 0.24 e Å^−^^3^
91 parameters	Δρ_min_ = −0.17 e Å^−^^3^
0 restraints	Extinction correction: *SHELXL97* (Sheldrick, 2008), Fc^*^=kFc[1+0.001xFc^2^λ^3^/sin(2θ)]^-1/4^
Primary atom site location: structure-invariant direct methods	Extinction coefficient: 0.031 (4)

### Special details

**Table:**

Geometry. All e.s.d.'s (except the e.s.d. in the dihedral angle between two l.s. planes) are estimated using the full covariance matrix. The cell e.s.d.'s are taken into account individually in the estimation of e.s.d.'s in distances, angles and torsion angles; correlations between e.s.d.'s in cell parameters are only used when they are defined by crystal symmetry. An approximate (isotropic) treatment of cell e.s.d.'s is used for estimating e.s.d.'s involving l.s. planes.
Refinement. Refinement of *F*^2^ against ALL reflections. The weighted *R*-factor *wR* and goodness of fit *S* are based on *F*^2^, conventional *R*-factors *R* are based on *F*, with *F* set to zero for negative *F*^2^. The threshold expression of *F*^2^ > σ(*F*^2^) is used only for calculating *R*-factors(gt) *etc*. and is not relevant to the choice of reflections for refinement. *R*-factors based on *F*^2^ are statistically about twice as large as those based on *F*, and *R*- factors based on ALL data will be even larger.

### Fractional atomic coordinates and isotropic or equivalent isotropic displacement parameters (Å^2^)

**Table:**

	*x*	*y*	*z*	*U*_iso_*/*U*_eq_	
O1	0.74430 (17)	0.55206 (10)	0.09878 (9)	0.0459 (4)	
O2	1.06414 (18)	0.53353 (12)	0.12399 (11)	0.0604 (4)	
O8	0.6552 (3)	0.7500	−0.08400 (12)	0.0550 (5)	
C1	0.6076 (3)	0.65913 (15)	−0.02489 (13)	0.0479 (5)	
H1	0.5863	0.5928	−0.0619	0.057*	
C2	0.7802 (2)	0.64791 (14)	0.04233 (12)	0.0409 (4)	
H2	0.8972	0.6349	0.0042	0.049*	
C3	0.8160 (3)	0.7500	0.10391 (17)	0.0382 (6)	
H3	0.9552	0.7500	0.1193	0.046*	
C6	0.4243 (3)	0.69639 (16)	0.02399 (13)	0.0510 (5)	
H6	0.3285	0.6518	0.0496	0.061*	
C8	0.7082 (5)	0.7500	0.19833 (19)	0.0494 (7)	
C9	0.9005 (3)	0.50142 (15)	0.13532 (13)	0.0451 (4)	
C10	0.8443 (3)	0.40349 (17)	0.18986 (17)	0.0647 (6)	
H10A	0.9539	0.3554	0.1947	0.097*	
H10B	0.7395	0.3669	0.1581	0.097*	
H10C	0.8029	0.4246	0.2521	0.097*	
H8	0.749 (3)	0.8125 (16)	0.2358 (16)	0.071 (7)*	
H81	0.575 (5)	0.7500	0.194 (3)	0.092 (12)*	

### Atomic displacement parameters (Å^2^)

**Table:**

	*U*^11^	*U*^22^	*U*^33^	*U*^12^	*U*^13^	*U*^23^
O1	0.0411 (6)	0.0396 (7)	0.0569 (8)	−0.0008 (6)	0.0002 (6)	0.0067 (6)
O2	0.0422 (8)	0.0569 (9)	0.0820 (10)	0.0018 (7)	0.0016 (7)	0.0071 (8)
O8	0.0746 (13)	0.0542 (11)	0.0362 (9)	0.000	−0.0022 (10)	0.000
C1	0.0580 (11)	0.0423 (10)	0.0434 (9)	−0.0048 (9)	−0.0061 (9)	−0.0032 (8)
C2	0.0420 (9)	0.0387 (9)	0.0420 (9)	−0.0002 (7)	0.0056 (8)	0.0006 (8)
C3	0.0330 (11)	0.0392 (13)	0.0425 (13)	0.000	0.0001 (10)	0.000
C6	0.0403 (9)	0.0602 (11)	0.0524 (11)	−0.0044 (8)	−0.0128 (8)	0.0003 (10)
C8	0.0507 (17)	0.0601 (18)	0.0374 (14)	0.000	0.0003 (13)	0.000
C9	0.0494 (10)	0.0372 (9)	0.0487 (10)	0.0021 (9)	0.0002 (8)	−0.0051 (9)
C10	0.0650 (13)	0.0516 (12)	0.0775 (15)	−0.0036 (11)	−0.0095 (12)	0.0119 (11)

### Geometric parameters (Å, °)

**Table:**

O1—C9	1.344 (2)	C3—C2^i^	1.547 (2)
O1—C2	1.444 (2)	C3—H3	0.9800
O2—C9	1.202 (2)	C6—C6^i^	1.318 (4)
O8—C1	1.432 (2)	C6—H6	0.9300
C1—C6	1.507 (2)	C8—H8	0.97 (2)
C1—C2	1.525 (2)	C8—H81	0.92 (4)
C1—H1	0.9800	C9—C10	1.481 (3)
C2—C3	1.547 (2)	C10—H10A	0.9600
C2—H2	0.9800	C10—H10B	0.9600
C3—C8	1.525 (4)	C10—H10C	0.9600
			
C9—O1—C2	116.98 (13)	C2—C3—H3	106.2
C1^i^—O8—C1	102.51 (19)	C2^i^—C3—H3	106.2
O8—C1—C6	102.74 (16)	C6^i^—C6—C1	107.70 (10)
O8—C1—C2	104.80 (15)	C6^i^—C6—H6	126.1
C6—C1—C2	113.07 (14)	C1—C6—H6	126.1
O8—C1—H1	111.9	C3—C8—H8	109.7 (13)
C6—C1—H1	111.9	C3—C8—H81	115 (2)
C2—C1—H1	111.9	H8—C8—H81	108.8 (18)
O1—C2—C1	106.53 (14)	O2—C9—O1	122.91 (17)
O1—C2—C3	112.28 (14)	O2—C9—C10	125.48 (18)
C1—C2—C3	113.59 (15)	O1—C9—C10	111.60 (16)
O1—C2—H2	108.1	C9—C10—H10A	109.5
C1—C2—H2	108.1	C9—C10—H10B	109.5
C3—C2—H2	108.1	H10A—C10—H10B	109.5
C8—C3—C2	114.47 (13)	C9—C10—H10C	109.5
C8—C3—C2^i^	114.47 (13)	H10A—C10—H10C	109.5
C2—C3—C2^i^	108.50 (19)	H10B—C10—H10C	109.5
C8—C3—H3	106.2		
			
C1^i^—O8—C1—C6	38.7 (2)	O1—C2—C3—C8	−30.9 (2)
C1^i^—O8—C1—C2	−79.64 (19)	C1—C2—C3—C8	90.0 (2)
C9—O1—C2—C1	154.76 (15)	O1—C2—C3—C2^i^	−160.12 (11)
C9—O1—C2—C3	−80.30 (19)	C1—C2—C3—C2^i^	−39.2 (2)
O8—C1—C2—O1	−175.93 (13)	O8—C1—C6—C6^i^	−24.28 (13)
C6—C1—C2—O1	72.93 (19)	C2—C1—C6—C6^i^	88.12 (14)
O8—C1—C2—C3	59.93 (19)	C2—O1—C9—O2	1.2 (3)
C6—C1—C2—C3	−51.2 (2)	C2—O1—C9—C10	−178.85 (15)

Symmetry codes: (i) *x*, −*y*+3/2, *z*.

### Hydrogen-bond geometry (Å, °)

**Table:**

*D*—H···*A*	*D*—H	H···*A*	*D*···*A*	*D*—H···*A*
C6—H6···O2^ii^	0.93	2.55	3.482 (2)	178

Symmetry codes: (ii) *x*−1, *y*, *z*.
